# Effectiveness of Recombinant Human Bone Morphogenetic Protein‐2 in Socket Preservation: A Randomized Controlled Clinical and Sequential Human Histological Trial (BMP‐2 TRIAL)

**DOI:** 10.1002/cre2.70134

**Published:** 2025-05-19

**Authors:** Saravanan Sampoornam Pape Reddy, Delfin Lovelina Francis, Harshini Thirumoorthi, Rahul, Manish Rathi, Harjeet Singh, Shaswata Karmakar, Shaili Pradhan

**Affiliations:** ^1^ Department of Periodontology Army Dental Centre (Research & Referral) New Delhi India; ^2^ Saveetha Dental College & Hospitals Saveetha University, Saveetha Institute of Medical and Technical Sciences (SIMATS) Chennai Tamil Nadu India; ^3^ Arunai Medical College & Hospital Tiruvannamalai Tamil Nadu India; ^4^ 3 Corps Dental Unit, Army Dental Corps Indian Army India; ^5^ Department of Periodontology Manipal College of Dental Sciences, Manipal Academy of Higher Education Manipal India; ^6^ Department of Periodontology & Oral Implantology Kathmandu Medical College Public Limited Kathmandu Bagmati Nepal

**Keywords:** bone morphogenetic protein 2, bone regeneration, histological technique, regeneration, ridge augmentation

## Abstract

**Objectives:**

The aim of this study was to evaluate the effectiveness of recombinant human bone morphogenetic protein‐2 (rhBMP‐2) coated biphasic calcium phosphate (BCP) in socket preservation in comparison to BCP.

**Material and Methods:**

Patients who underwent extraction of maxillary premolars were randomized to receive rhBMP‐2/BCP (*n* = 15) and BCP alone (*n* = 15). All sites were primarily closed using a pedicled connective tissue flap. Biopsy was carried out in patients at 3 (*n* = 10), 6 (*n* = 10), and 9 (*n* = 10) months.

**Results:**

At 3 months, BMP and non‐BMP groups had 3.17% and 3.12% new bone formation, respectively, and 3.8% and 37.85% of residual grafts, respectively, and 25.86% and 25.99% connective tissue component, respectively. At 6 months, both groups revealed 67.42% and 16.55% essential bone growth, 1.74% and 3.04% of residual graft, and 1.3% and 67% connective tissue component, respectively. At 9 months, the BMP group revealed 93.6% new bone formation, 0.68% residual graft, and 1.9% connective tissue component, while the non‐BMP groups had 23.35% new bone formation, 4.45% residual graft, and 44.06% connective tissue component.

**Conclusion:**

This study demonstrated that rhBMP‐2 coated BCP significantly enhanced early graft substitution in socket preservation sites compared to BCP alone. There was a significantly higher percentage of new bone formation in the rhBMP‐2/BCP group both at 6 and 9 months. Additionally, the rhBMP‐2/BCP group exhibited faster resorption of the graft material and earlier maturation of newly formed bone. These findings strongly suggest that rhBMP‐2/BCP can be an effective treatment modality for socket preservation, promoting predictable and accelerated bone regeneration.

## 1 Introduction

Preserving the volume of the alveolar ridge after tooth extraction is a crucial consideration in dentistry, especially when preparing for future prosthetic rehabilitation by implants. Reduced ridge dimensions can make implant surgery more difficult and necessitate complex surgical procedures. Several bone grafting methods and biomaterials have been studied to combat this challenge, aiming to promote the growth of new bone and maintain the alveolar ridge dimensions. Various biomaterials have yielded diverse outcomes (Canullo et al. 2022). The efficacy of bone replacement grafts depends on the effective replacement of the graft material with newly formed bone at an equivalent pace of graft resorption kinetics. Currently, there is no biomaterial that has a substitution rate equal to that of a bone replacement graft (Atieh et al. 2021).

An emerging method that has attracted interest in scientific literature is the utilization of recombinant human bone morphogenetic protein‐2 (rhBMP‐2) in various orthopedic procedures (Jeong et al. 2015). rhBMP‐2 is a highly effective factor that can induce the growth of new bone and help maintain the structure of the alveolar ridge. Nevertheless, there is a scarcity of literature, that demonstrates the specific characteristics and quality of bone, that forms following augmentation with rhBMP‐2. The duration required for the development of mature lamellar bone by rhBMP‐2 remains uncertain. Hence, this study investigated the effectiveness of rhBMP‐2 at three different timelines when used together with biphasic calcium phosphate (BCP), as a grafting material for preserving the alveolar ridge after tooth extraction. The study utilized a rigorous sequential approach, which involved standardized surgical techniques, sequential histological analysis of bone core biopsies at three different time points and quantitative histomorphometry.

## 2 Materials and Methods

### 2.1 Study Design

The study was a randomized, active‐controlled clinical trial performed at a postgraduate teaching institution. The study employed a parallel arm design with single blinding, only of the subjects and was conducted in a tertiary care teaching institution. The duration of the study was from Sep 2016 to Dec 2019 and obtained ethical clearance from the Institutional Ethical Clearance (DP/ADCRR/01/2016). Since the study was started in 2016, no separate clinical trial registration was done. The study complied with the guidelines of the Declaration of Helsinki, 2013. The study was conducted in compliance with CONSORT guidelines.

### 2.2 Subjects

The desired number of participants in each group was 15 (*n* = 24), determined by the minimum detectable effect size reported in a prior published study. Based on an attrition rate of 20% (*n* = 3), a total sample size of 30 was determined, with 15 participants in each arm. The significance level was established at 5% and power of the study at 80%. Out of a total of 245 individuals, 215 were eliminated after screening, while 30 who met the criteria were enrolled. The inclusion criteria encompassed individuals who displayed indications for the removal of maxillary premolars with intact buccal and palatal plates, teeth with no pre‐existing periodontal or periapical infections were included to avoid confounding factors that could affect healing dynamics and bone regeneration, with good overall health, and were between the ages of 20 and 45, regardless of gender. The study excluded individuals who had clinical and radiological signs of localized infection, those who refused to undergo bone core biopsy, and pregnant or lactating women, as well as individuals who were on long‐term systemic medications. The sites with socket morphology compromised by trauma, infections, or pre‐existing pathological lesions were excluded in addition to patients presenting with adjacent teeth showing periodontal or periapical involvement to minimize potential cross‐contamination of the graft site. Extraction sockets were assessed clinically and radiographically to confirm intact socket walls before inclusion. Only sockets with a Class I socket were included to standardize the baseline morphology for all study participants. The sampling methodology employed was simple random sampling without replacement. The study CONSORT flow chart is presented in Figure S1. The approach employed for randomization was block randomization. Following the randomization process, the subjects were randomized into either the rhBMP‐2 with BCP group (test) or the BCP only (control) group with 1:1 allocation ratio. S.P. enrolled the participants, D.F. prepared the random allocation sequence.

### 2.3 Graft Materials

The sequentially numbered container of graft materials utilized in the present study consisted of rhBMP‐2 combined with BCP (Cowell BMP, Korea) as the experimental material and BCP (BoneSigma BCP, Sigmagraft, United States) alone as the control material. The quantity of graft material required for each case was contingent upon the size of the extraction socket. In the majority of cases, the quantity of graft materials used for grafting varied between 1.5 and 2.0 g. The dosage of rhBMP‐2 ranged from 1.0 to 2.0 mg, depending on the quantity of graft material utilized.

### 2.4 Surgical Procedure

**Figure 1:**
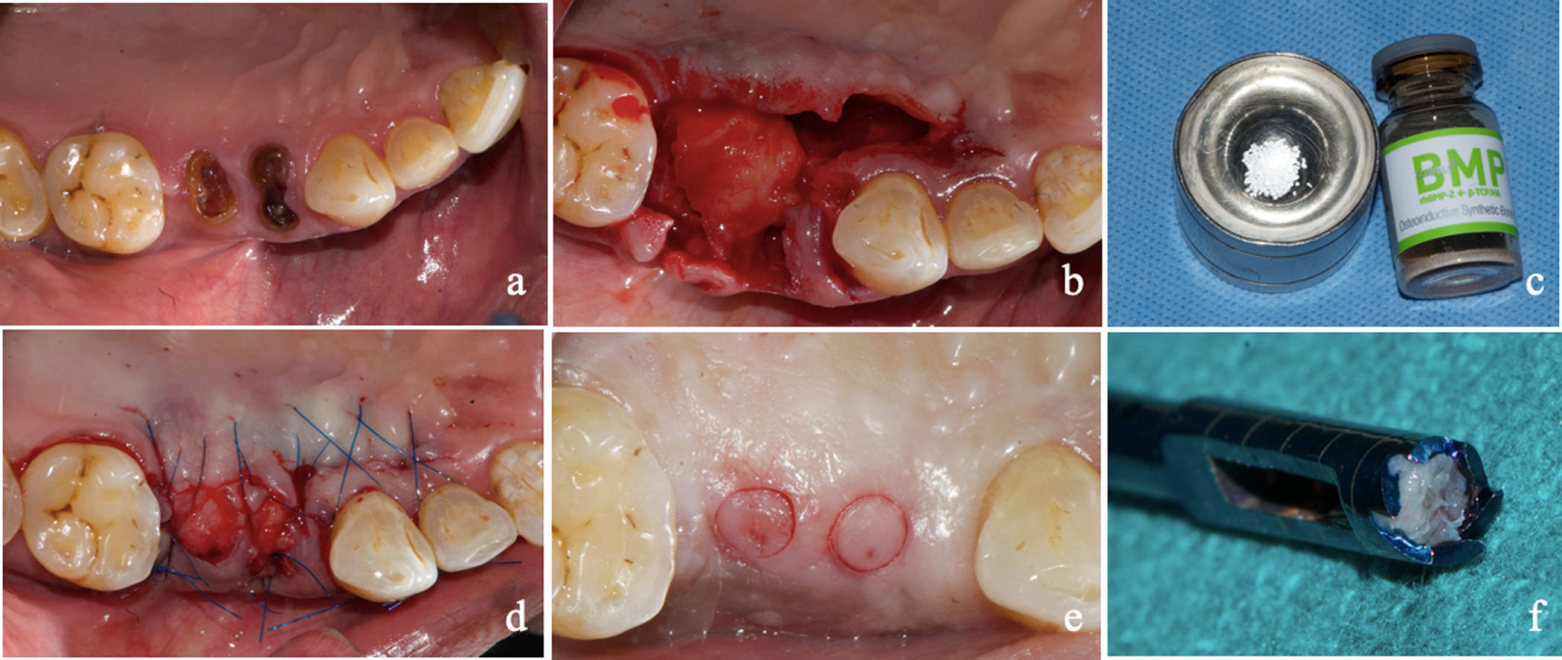
(a) Preoperative clinical situation showing root stumps in relation to maxillary premolars. (b) Preparation of rotated connective tissue pedicle post‐extraction. (c) rhBMP‐2 with BCP biomaterial which was used. (d) Flap closure utilizing the rotated connective tissue pedicle for wound coverage. (e) Punch biopsy at second stage during implant placement. (f) Bone core biopsy (2 x 6 mm) sample obtained.

A single proficient periodontist executed the surgical procedure to guarantee consistency. Following a 4‐week period of initial therapy, patients underwent socket preservation under local anesthesia. Briefly, a flapless extraction was employed and the socket was meticulously debrided with a bone curette and thereafter filled with test/control material. A rotated palatal pedicle flap was used to close the site, effectively covering the extraction socket. The flap was fixed in place using 5/0 prolene suture. All patients were prescribed analgesics postoperatively (Figure 1).

### 2.5 Bone Core Biopsy

The two patient groups were randomly assigned to undergo bone core biopsy at one of three distinct intervals: 3 months (*n* = 5 in test and 5 in control), 6 months (*n* = 5 in test and 5 in control), or 9 months (*n* = 5 in test and 5 in control), for sequential histomorphometric analysis. A surgical trephine was employed to perform a 2 mm bone core biopsy immediately before implant osteotomy. The samples were fixed in 10% formalin and subsequently processed for histological examination.

### 2.6 Histomorphometry

**Figure 2:**
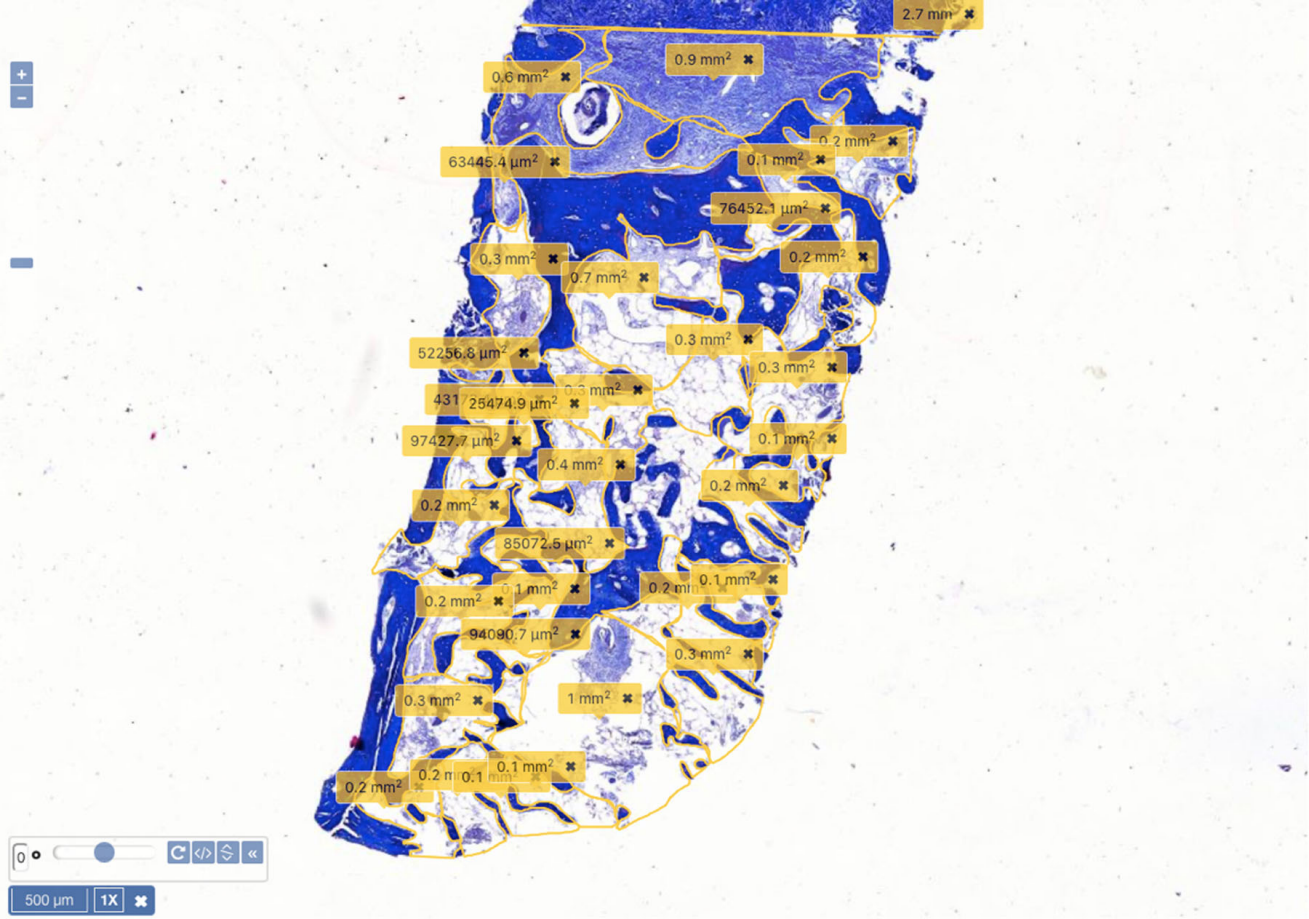
Histomorphometric analysis and measurements in a representative sample showing the measurement values of connective tissue component using Pathomation software v1.0 (×1 magnification, Alcian Blue).

Histomorphometric analysis was conducted by a board‐certified oral pathologist using Pathomation software v1.0 (Belgium) after staining 4 μm sections. The histomorphometric analysis was conducted in accordance with the methodology previously described by Kim et al. (2015). Evaluation of histomorphometric parameters, included the total area of newly formed bone (NB) as primary outcome, residual graft (RG) total area as secondary outcome, and connective tissue (CT) component (Figure 2). In addition to area measurements in μm^2^, all three parameters were analyzed in relation to the total area of the biopsied tissue and expressed as a percentage. The histological sections were independently analyzed by an additional examiner in addition to the oral pathologist who calculated the histomorphometric measurements. The two examiners ultimately reached a consensus. The trial outcome measures remained unaltered subsequent to the trial's commencement.

### 2.7 Statistical Analysis

Descriptive statistics were implemented on the demographic information (Table S1). The proportion of male and female subjects was 43.33% (*n* = 13) and 56.66% (*n* = 17), respectively. The mean age of male and female participants was 44.85 ± 7.18 and 45.15 ± 6.89, respectively. The data that was collected was subjected to the normality assumption of inferential statistics. The Kruskal–Wallis test was implemented due to the non‐normal distribution of the data. The test and control groups were compared at three, six and nine months in terms of the newly formed bone, residual graft, and soft tissue components. Intragroup comparisons were implemented to evaluate the disparity in primary and secondary outcome measures between 3 and 6 months, 3 and 9 months, and 6 and 9 months.

## 3 Results

### 3.1 Intragroup Comparisons

**Figure 3:**
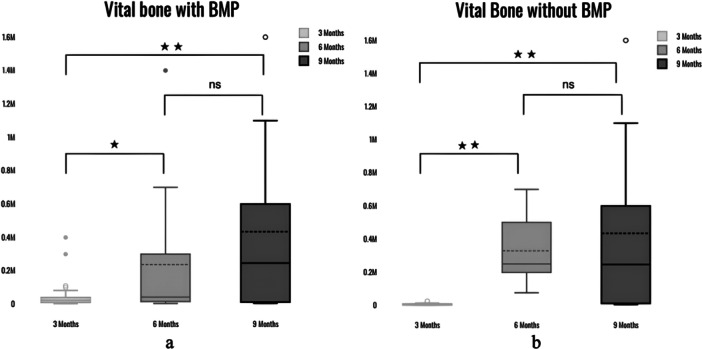
(a) Box and Whisker plot showing differences In Vital bone formation in BMP group at three different timelines. (b) Box and Whisker plot showing differences In Vital bone formation in non‐BMP group at three different timelines.

**Figure 4:**
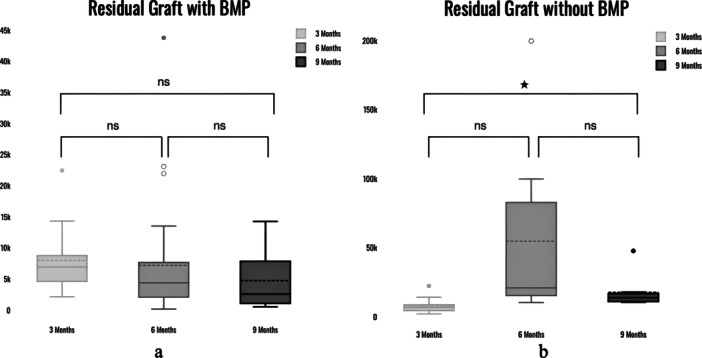
(a) Box and Whisker plot showing differences in residual graft in BMP group at three different timelines. (b) Box and Whisker plot showing differences in residual graft in non‐BMP group at three different timelines.

**Figure 5:**
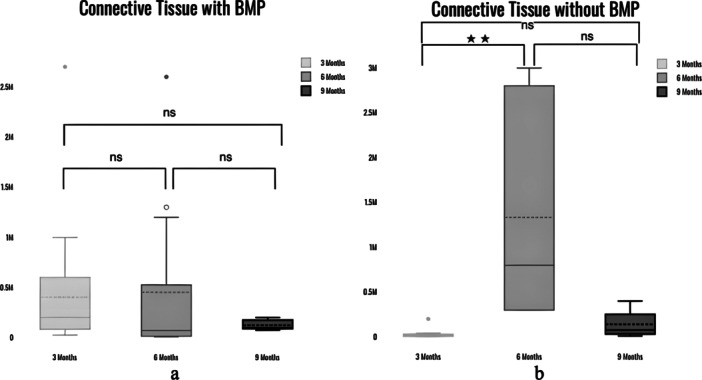
(a) Box and Whisker plot showing differences in connective tissue in BMP group at three different timelines. (b) Box and Whisker plot showing differences in connective tissue in non‐BMP group at three different timelines.

With BMP, the difference in new bone (NB) between 3 and 6 months, between 6 and 9 months was statistically significant with *p*‐value < 0.05 (*p* = 0.02), and *p* = 0.002, respectively. There was no statistically significant difference between NB at 6 and 9 months with *p* = 0.16. Without BMP, the difference in NB between 3 and 6 months, between 6 and 9 months was statistically significant with *p*‐value < 0.05 (*p* = 0.0004), and *p* = 0.0005, respectively. There was no statistically significant difference between NB at 6 and 9 months with *p* = 0.584 (Figure 3). The residual graft (RG) with BMP at 3 vs. 6 months did not have a statistically significant difference with a *p*‐value of 0.09, whereas between 6 and 9 months, it was 0.05. There was no statistically significant difference for RG between 3 and 9 months with *p*‐value of 0.6. The RG without BMP at 3 vs. 6 months did not have a statistically significant difference with a *p*‐value of 0.2, whereas between 6 and 9 months, it was *p* = 0.06. There was a statistically significant difference between 3 and 9 months with *a p*‐value of 0.004 (Figure 4). The Kruskal–Wallis test indicated that there was no statistically significant difference in BMP group in the connective tissue (CT) between 3 and 6 months (*p* = 0.09), between 3 and 9 months (*p* = 0.38) and between 6 and 9 months (*p* = 0.67). There was statistically significant difference in CT without BMP group between 3 and 6 months with *p* = 0.0001. There was no significant difference between groups at 3 and 9 months (*p* = 0.11) and between 6 and 9 months (*p* = 0.14) for CT (Figure 5). Further, there was no significant difference in bone formation between the BMP and non‐BMP groups at 6 and 9 months. Furthermore, the presence of rhBMP‐2 resulted in much lower RG at all time periods, indicating more rapid graft resorption and replacement with NB. This was most noticeable 6 months after surgery, implying a minimum 6‐month waiting period before implant placement in rhBMP‐2‐grafted sites. The adverse effects were modest, limited to intra‐oral edema at the rhBMP‐2 grafted sites (*n* = 15, 100%), and were well tolerated by all patients. The amount of CT was also significantly lower in the rhBMP‐2 group at early time points.

### 3.2 Intergroup Comparisons

There was a statistically significant difference with or without BMP in NB at 3 months with *p*‐value = 0.04. However, the percentage of NB with or without BMP did not differ significantly at 6 months and at 9 months with *p* = 0.58 and *p* = 0.67, respectively. With Kruskal–Wallis non‐parametric test, the RG at 3 months and 6 months with or without BMP did differ significantly with a *p*‐value of 0.004 and 0.0003, respectively. The RG also exhibited a statistically significant difference at 9 months, with a *p*‐value of 0.0006. The amount of CT at 3 months with or without BMP did differ significantly with a p‐value of 0.019, whereas at 6 and 9 months, it did not differ significantly with *p*‐value of 0.07 and 0.7, respectively. The percentage of NB at 3 months was 3.17% and 3.12% with and without rhBMP‐2, respectively. The percentage of RG at 3 months was 3.8% and 37.85% with and without rhBMP‐2, respectively. The percentage of CT at 3 months was 25.86% and 25.99% with and without rhBMP‐2, respectively. The percentage of NB at 6 months was 67.42% and 16.55% with and without rhBMP‐2, respectively. The percentage of RG at 6 months was 1.74% and 3.04% with and without rhBMP‐2, respectively. The percentage of CT at 6 months was 1.3% and 67% with and without rhBMP‐2, respectively. The percentage of NB at 9 months was 93.6% and 23.35% with and without rhBMP‐2, respectively. The percentage of RG at 6 months was 0.68% and 4.45% with and without rhBMP‐2, respectively. The percentage of CT at 9 months was 1.9% and 44.06% with and without rhBMP‐2, respectively (Table 1).

**Table 1:** Comparison of study parameters at different time points.

Parameter	With rhBMP2^a^ – percentage (SD)^b^	Mean area ± SD (μm^2^)	Without rhBMP2 – percentage (SD)	Mean area ± SD (μm^2^)
At 3 months				
New Bone	3.12% (1.8)	40271.6 ± 65882.3	3.17% (1.45)	7010.1 ± 6344.1
Residual Graft	3.8% (2.4)	8042 ± 5538.9	37.85% (12.45)	77473.583 ± 75708.527
Connective Tissue	25.86% (20.5)	403068.0 ± 545790.9	25.99% (18.25)	31510.5 ± 51997.7
At 6 months				
New Bone	67.42% (30.77)	237660.5 ± 370824.9	16.55% (10.38)	329446 ± 230074.4
Residual Graft	1.74% (1.3)	7234.9 ± 9573.4	3.04% (2.6)	54922.7 ± 60569.5
Connective Tissue	1.3% (0.9)	7234.9 ± 9573.4	67.00% (34.65)	1333333.3 ± 1235583.5
At 9 months				
New Bone	93.6% (2.8)	580434.3 ± 853268.8	23.35% (16.48)	434243.5 ± 544045.1
Residual Graft	0.68% (0.35)	4796.8 ± 4276.8	4.45% (3.82)	17837.1 ± 12398.3
Connective Tissue	1.90% (1.28)	122626.4 ± 53518.4	44.06% (28.45)	141210.5 ± 176278.4

^a^ Recombinant human bone morphogenetic protein‐2.

^b^ SD, standard deviation.

## 4 Discussion

The objective of the present investigation was to evaluate the effectiveness of rhBMP2 loaded on BCP in socket preservation through sequential histomorphometric analysis at 3, 6, and 9 months. The efficacy of *Escherichia coli* rhBMP‐2 was assessed in a preclinical study, which concluded that it is comparable to autogenous grafts (Nosho et al. 2022). Furthermore, there are additional studies that assessed the efficacy of rhBMP‐2 by assessing radiographic density (Boyne and James 1980). Nevertheless, histomorphometric analysis is the gold standard for evaluating the NB formation and is more effective than noninvasive methods. In this study, histology samples were obtained during implant placement at 3, 6, and 9 months. However, we abstained from evaluating the implant's success or failure in these patients because it was beyond the scope of this study. In this study, commercially available BCP particles coated with rhBMP‐2 were utilized. The same biomaterial resulted in successful bone regeneration following maxillary sinus lift procedures (Susin et al. 2018; Lee et al. 2015).

Multiple animal investigations have produced histological data indicating the osteoinductive and osteoconductive properties of rhBMP‐2 (Kim et al. 2018). Human studies have shown that rh‐BMP‐2 resulted in the development of a comparable amount of essential bone to the gold standard, autogenous bone (Torrecillas‐Martinez et al. 2013). A thorough assessment has also shown that rhBMP‐2 in an absorbable collagen sponge (ACS) is a feasible alternative to autogenous bone grafts in maxillary sinus augmentation. In socket preservation, the socket dimensions were preserved, while the ridge width was increased using rhBMP‐2 (Freitas et al. 2015). BCP is an effective carrier due to its inherent characteristics that facilitate ectopic bone regeneration by modulating T regulatory cells, hence it was used as a control (Li et al. 2024). Utilizing BCP as a carrier for rhBMP‐2 allows for the maintenance of graft volume and the provision of mechanical stability until the development of mature lamellar bone. The utilization of this combination resulted in a substantial improvement in the preservation of alveolar ridge width and height over a 12‐week period in a previous clinical trial (Han et al. 2021). However, it did not incorporate a sequential histological analysis. A non‐inferiority trial found no statistically significant difference in the preservation of the alveolar ridge between rhBMP‐2 and ACS or β‐TCP (Jo et al. 2019). Interestingly, rhBMP‐2 led to regeneration of buccal plate at dehiscence sites, with a significant difference between the test group (4.75 mm) and the control group (1.85 mm) (Coomes et al. 2014).

The optimal dosage of rhBMP‐2 has not yet been determined, and a suitable carrier with sustained release qualities has not been observed to date. The radiographic densitometric analysis revealed that a modest dosage of rhBMP‐2 effectively stimulated bone regeneration within a 6‐week period (Sun et al. 2023a). Clinicians can anticipate a broad spectrum of interindividual variability in bone formation on account of the variability in the response to rhBMP‐2. However, a systematic review with a high risk of bias and heterogeneity concluded that 1.5 mg/mL of rhBMP‐2 was more effective in preserving extraction sockets (Avila‐Ortiz et al. 2019).

It is generally estimated that 1 gram of autogenous bone contains 2 ng of BMP, as naturally occurring BMP concentrations in autogenous bone are typically low and vary based on factors such as the source of the autogenous bone and the individual's health (Schwartz et al. 1998). In contrast, rhBMP‐2 can be incorporated into graft materials at significantly higher concentrations to enhance their osteoinductive potential (Wikesjö et al. 1999). For example, clinical applications of rhBMP‐2 in periodontal regeneration commonly use concentrations between 0.5 mg/mL and 1.5 mg/mL, demonstrating substantial efficacy in promoting bone formation. While higher concentrations of BMPs in graft materials can promote accelerated bone healing, they also pose risks such as localized inflammation, soft tissue swelling, and, in some cases, complications such as ectopic bone formation (Carragee et al. 2011). Therefore, the clinical use of BMPs, especially recombinant forms, requires careful consideration of their dosage and potential side effects to balance the benefits of enhanced bone regeneration against possible adverse effects. The principal action is to cause cell migration with lamellipodia. The stationary cells convert into polarized cells, which are activated by the production of filopodial extensions, cell protrusions, and nuclear effectors and transcription factors. It also has effects on mesenchymal stem cells (MSC) by adhering to their membrane and promoting the activation of nuclear genes. It induces the differentiation of MSCs into osteoblasts, which promotes bone production. However, it interacts with other differentiation signals, accelerating the differentiation of many cellular phenotypes. This behavior is similar to the mesenchymal phenotypes observed during In Vivo bone growth (Kathami et al. 2024; Zhu et al. 2020; Zhao 2003). Research has shown that rhBMP‐2 can increase the production of BMPs in osteoclasts, facilitating the recruitment and differentiation of osteoblasts. Non‐transcriptional processes involve the reorganization of the cytoskeleton in cells by altering the actin structure, which operates via both SMAD and non‐SMAD pathways (Li et al. 2019; Zhang et al. 2016). This causes a rapid rise in osteoblasts, fibroblasts, and keratinocytes. Among several factors, the Twist‐2 transcriptional factor promotes tissue regeneration in osseous tissues, such as extraction sockets.

While rhBMP‐2 may not be as effective as autogenous grafts, it does reduce donor site morbidity. In socket preservation, it emerged to lead much greater bone formation after 6 weeks than the other graft materials as in agreement with the present study (Sun et al. 2023b). The need of maintaining a continuous and regulated release of BMP‐2 is emphasized to ensure the optimum potential stimulation of bone growth (Liu et al. 2007). In this study, it is unclear if the rhBMP‐2 is adsorbed or coated over the BCP particles. Further research is needed to demonstrate long‐term safety, fine‐tune the dosage, and develop more effective carriers. The primary finding in terms of safety is that, the use of a low‐dose protocol of rhBMP‐2 significantly reduces complications (Kim et al. 2019; Mendenhall et al. 2021). Therefore, in this investigation, a minimal dosage of rhBMP‐2 was administered and no substantial adverse events were documented. The current evidence supporting the use of rhBMP‐2 in craniofacial surgery is limited and shows varied results. rhBMP‐2 was suggested as a potential treatment for alveolar ridge augmentation; however, it also underscores that the evidence supporting its use in maxillary sinus augmentation is insufficient and discourages its use in calvarial reconstruction (Ramly et al. 2019). Furthermore, because there is insufficient data on the long‐term effects of rhBMP‐2 on craniofacial growth, its use in patients under the age of 18 remains off‐label. As a result, the study only included adult patients.

The histological data suggest that, rhBMP‐2 considerably improves bone regeneration in distracted alveolar bone after 6 weeks, but the current study required 6–9 months for maximum bone regeneration (Terbish et al. 2015). Localized low‐dose rhBMP‐2 was efficacious in promoting bone regeneration in a mandibular segmental defect study, with statistically significant differences between the treatment and control groups (Carlisle et al. 2019). However, the same has demonstrated that, the use of BMPs for periodontal bone regeneration has the potential to increase the incidence of ankylosis (Muthukuru 2013). In another study, using rhBMP‐2 with or without a membrane produced different outcomes depending on the time point. At 12 weeks, the presence or absence of a membrane had no meaningful effect on new bone formation (Jones et al. 2006); hence, no membrane was used in this study.

The combination of rhBMP‐2 with additional therapeutic techniques such as gene therapy or stem cell therapy has the potential to improve the efficiency of bone regeneration. Future studies with larger sample size should focus on personalized rhBMP‐2 therapy based on patient characteristics such as age, genetics, and kind of osseous defect (Carreira et al. 2014). The present study revealed significant localized soft tissue swelling as an unfavorable effect, which was induced by the inherent tendency of BMP‐2 to absorb physiological fluids, which was consistent with earlier findings (Lubelski et al. 2015). The intraoral edema decreased after a 3‐week period, which is consistent with the findings of other published studies (Woo 2012). Additional undesirable occurrences recorded in the literature include the development of seromas and cystic lesions, with the potential but unconfirmed risk of developing cancer (Skovrlj et al. 2015). The trial limitations include low statistical power, which resulted in a small sample size, non‐evaluation of radiographic density and the presence of BMP antibodies in blood. It is crucial to note that, this study is a histological wound healing trial, not an implant study, despite the fact that all subjects received dental implants. This study demonstrated that a minimum of 6 months before implant placement is required to obtain a considerable amount of mature bone growth after rhBMP‐2 in socket preservation. The amount of new bone formation can be increased by delaying it for up to 9 months.

This study investigated the impact of rhBMP‐2 loaded on BCP on the production of NB in socket preservation when compared to BCP alone. The results indicated that rhBMP‐2 significantly enhances initial bone formation. Utilizing rhBMP‐2 in socket preservation has the potential to result in better clinical results for patients, such as shorter healing time and greater bone regeneration. The study findings indicate that rhBMP‐2 plays a crucial role in stimulating bone regeneration, leading to faster and more effective replacement.

## Author Contributions

Saravanan Sampoornam Pape Reddy conceived the ideas, conceptualization, and methodology. Delfin Lovelina Francis performed the study design, blinding, and sampling. Harshini Thirumoorthi prepared the draft manuscript and performed the histomorphometry. Rahul performed the data collection. Manish Rathi performed the repeat histomorphometry and validation. Harjeet Singh performed the data analysis. Shaswata Karmakar performed the formal analysis. Shaili Pradhan led the project administration and final manuscript review.

## Ethics Statement

It is declared that this study obtained ethical committee clearance vide application no DP/ADCRR/09/2023 from the Army Dental Centre (Research & Referral), New Delhi, India.

## Consent

All the participants of this study have signed the written informed consent in their regional language.

## Conflicts of Interest

The authors declare no conflicts of interest.

## Permission to Reproduce Material From Other Sources

Not applicable as none of the material was reproduced from any sources.

## Supporting information

SUPPLEMENTARY.

CONSORT‐2010‐Checklist.

Flowchart.

## Data Availability

The data that support the findings of this study are available from the corresponding author upon reasonable request.
