# Supplementary material for: Effectiveness of Recombinant Human Bone Morphogenetic Protein‐2 in Socket Preservation: A Randomized Controlled Clinical and Sequential Human Histological Trial (BMP‐2 TRIAL)
Source: Clin Exp Dent Res. 2025 May 19;11(3):e70134. doi: 10.1002/cre2.70134 (PMC12087511; doi:10.1002/cre2.70134)
Supplement: Supplementary file 1 — SUPPLEMENTARY. [file CRE2-11-e70134-s002.docx]

**SUPPLEMENTARY TABLES / FIGURES**

**SUPPLEMENTARY TABLE 1**

Demographic distribution of study participants

|  | BMP/BCP  (n=15) | BCP Only  (n=15) | Total  (n=30) |
| --- | --- | --- | --- |
| Sex, n (%) | | | |
| Male | 7 (46.66) | 6 (40%) | 13 (43.33%) |
| Female | 8 (53.33) | 9 (60%) | 17 (56.66%) |
| Age, Years | | | |
| Mean | 44.85 | 45.15 | 45.0 |
| SD | 7.18 | 6.89 | 7.03 |

BMP-Bone morphogenetic protein, BCP-Biphasic calcium phosphate, n=number of participants

**SUPPLEMENTARY FIGURE LEGENDS**

SUPPLEMENTARY FIGURE 1 – Study flowchart as per CONSORT diagram.
