# Supplementary figures and images for: Effectiveness of Recombinant Human Bone Morphogenetic Protein‐2 in Socket Preservation: A Randomized Controlled Clinical and Sequential Human Histological Trial (BMP‐2 TRIAL)
Source: Clin Exp Dent Res. 2025 May 19;11(3):e70134. doi: 10.1002/cre2.70134 (PMC12087511; doi:10.1002/cre2.70134)

**CONSORT FLOW CHART**


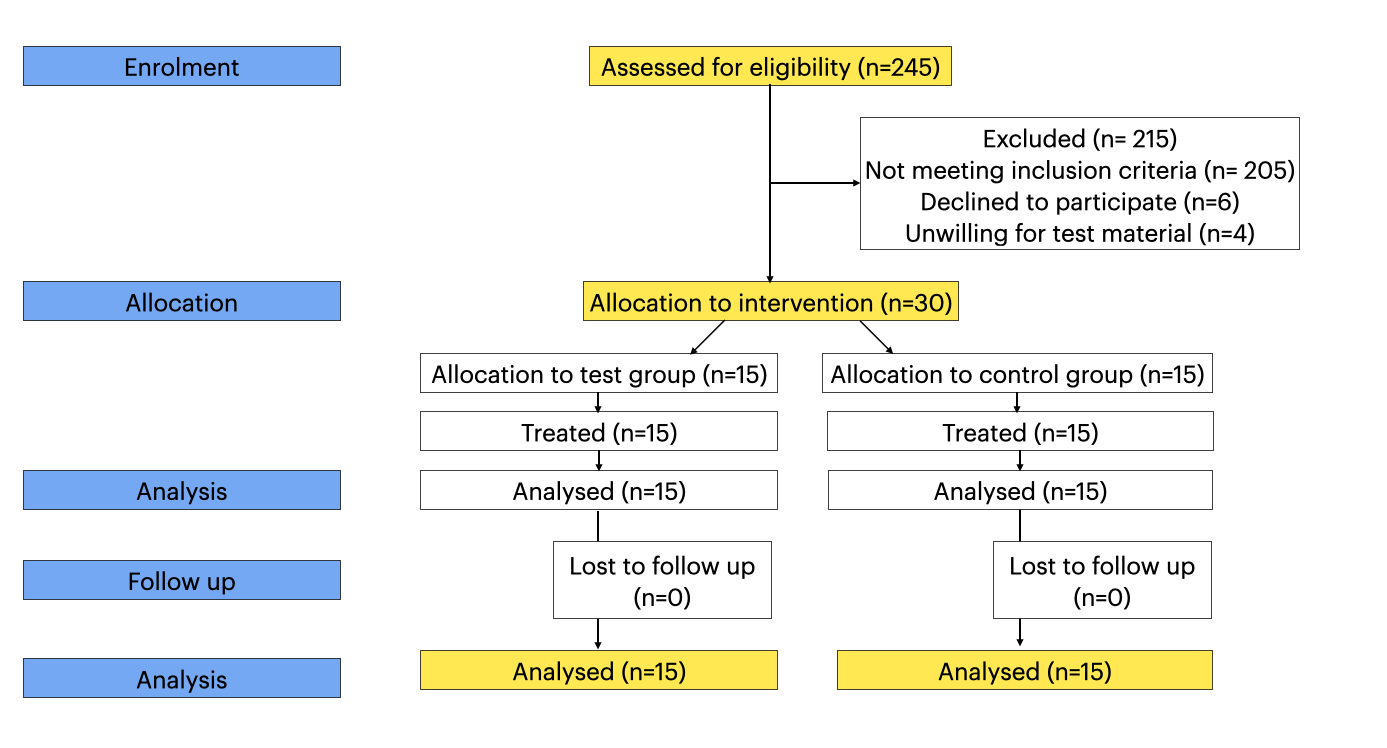

Supplement: Supplementary file 3 — Flowchart. [file CRE2-11-e70134-s003.docx]
